# Recent advances in biomass valorization through thermochemical processes, bio-oil production and AI strategies: a concise review

**DOI:** 10.1039/d5ra05770a

**Published:** 2025-11-24

**Authors:** Yasmeen Saleh, Labeeb Ali, Mohammednoor Altarawneh

**Affiliations:** a United Arab Emirates University, Department of Chemical and Petroleum Engineering Sheikh Khalifa bin Zayed Street Al-Ain 15551 United Arab Emirates labeeb_ali@uaeu.ac.ae; b Abu Dhabi Polytechnic University, Petroleum Engineering Technology Department Abu Dhabi 111499 United Arab Emirates mn.altarawneh@uaeu.ac.ae

## Abstract

Biomass valorization through thermochemical pathways is an appropriate option to generate bioenergy, reducing the reliance on petroleum-based fuels and minimizing their adverse environmental impacts. Biomass waste accumulates abundantly worldwide and is typically managed through landfilling and incineration, posing threats to the environment. Alternatively, biomass waste can be converted into valuable products through thermochemical techniques. This review provides an overview on the categories of biomass and the most widely utilized thermochemical pathways for its conversion, including pyrolysis, gasification, liquefaction, and combustion. Additionally, the characteristics and conditions of the processes, configuration of the utilized reactors, the main products, bio-oil catalytic upgrading, advanced upgrading techniques, and commercial-scale thermochemical plants are discussed. Moreover, it summarizes the main findings of the notable studies that investigated the thermal degradation of various biomass types. Furthermore, it highlights the role of artificial intelligence (AI) in forecasting bioenergy production from lignocellulosic biomass. Finally, the main challenges in the thermochemical valorization of biomass, which necessitate further research, are identified.

## Abstract 2

A critical review of the recent advances pertinent to the thermochemical conversion of biomass, including the process characteristics and the utilized reactor configurations, emphasising on the products upgrading with AI applications.

## Graphical abstract

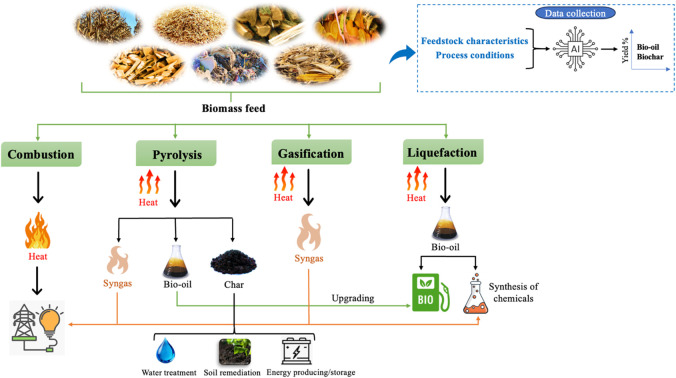

## 1. Introduction

The tremendous rise in fossil fuel consumption over the past few decades due to the rapid population growth and industrialization has led to the depletion of petroleum-based fuel resources and irrevocable impacts on the environment, particularly the emission of greenhouse gases. As a result, several countries are now actively working to adopt sustainable and renewable energy sources to mitigate the climate change crisis and shift their dependency on conventional fuel resources.^1–3^ According to the International Energy Agency (IEA), it is expected that the global demand for biofuel will grow by 41 billion liters by 2026.^4^ Biomass is a term that describes the organic material that originates from plants *via* the photosynthesis process;^2^ it is a renewable energy source that has the ability to be converted into biofuel and chemicals through the thermal breakdown of lignocellulosic materials.^5^ Biomass is a promising alternative to petroleum-based fuels due to its availability and carbon neutrality, spanning diverse resources such as agricultural waste, food processing waste, energy crops, municipal solid waste, and forestry waste.^6–8^ The aforementioned categories can be transformed into bio-oil, syngas, and biochar through thermal degradation, in addition to valuable products, such as ethanol, furfural, and phenol.^9,10^ The major thermochemical valorization processes include pyrolysis, gasification, combustion, and liquefaction.^11^ Combustion is the only process that produces heat, which can be used directly for electricity generation, while the pyrolysis, gasification, and liquefaction techniques demand energy input to yield bioenergy. In addition to the generation of biofuel, the thermal conversion of biomass has the potential to produce valuable chemicals, like plastics, fertilizers, and pharmaceuticals, that have applications in several industries.^12–14^ The end products of thermochemical conversion depend principally on the treatment process, operating conditions, and biomass feedstock composition.^5^ Artificial intelligence (AI), which focuses on developing models capable of learning from datasets and making decisions, is widely utilized in various industrial sectors. Biomass thermochemical conversion for bioenergy production is one of the sectors that can employ AI algorithms to enhance its performance and productivity.^15^

The aim of the present review is to discuss the major biomass categories and the general characteristics of the main thermochemical techniques applied for biomass conversion. Moreover, it provides an overview of the commonly used reactors, the characteristics of the products (bio-oil, pyrolysis gas, and biochar), bio-oil upgrading, and the role of AI in biomass conversion, while also summarizing notable studies on biomass valorization. In addition, the main challenges associated with biomass conversion through thermochemical techniques are discussed.

## 2. Biomass categories

**Fig. 1:**
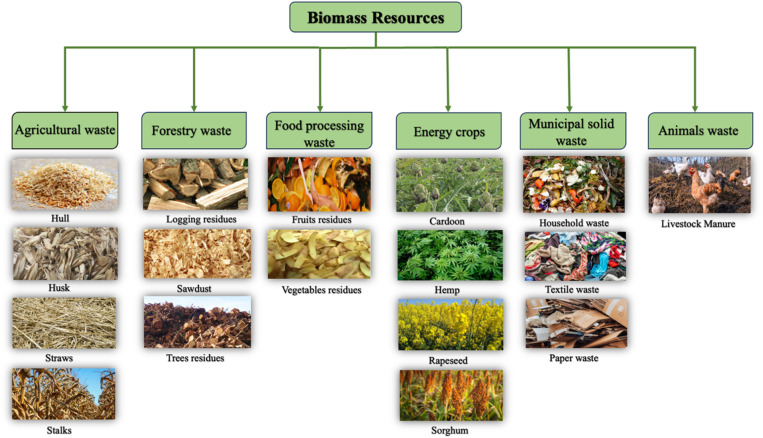
Major biomass categories and some examples.

Biomass is an inclusive term that describes the organic matter produced by the photosynthesis process, existing in the form of plants and microorganisms.^1^ The solar energy stored in plants is called biomass energy, which can be recovered by thermochemical processes.^8^ In the photosynthesis process, plants absorb water, CO_2_, and solar energy from the atmosphere to produce oxygen and monosaccharides such as glucose, which are further converted into secondary products such as polysaccharides, proteins, and lipids. The common categories of biomass are shown in Fig. 1, which include agricultural crop residues, forestry waste, food processing waste, energy crops, municipal waste, and animals waste.^6–8^ Annually, around 180 billion tons of lignocellulosic biomass are produced from agricultural and forestry residues, while only 8.2 billion tons are reused.^16,17^ Most of the produced biomass is managed *via* landfilling or incineration, posing adverse environmental impacts. Instead, lignocellulosic biomass can be valorized into a variety of products such as fuel substitutes, bio-plastics, fertilizers, chemicals, and construction materials.^17,18^

### 2.1. Agricultural waste

Significant quantities of crop residues are produced annually worldwide during harvesting or processing.^8^ The world's population rapid growth has increased the demands on crop production, further leading to an increase in the generated crops waste.^19^ These residues comprise straws, stalks, seeds, leaves, husks, *etc.*,^20^ which essentially consist of lignocellulosic materials. Annually, about 140 billion tons of agricultural waste are generated but only 40% is reused for fuel production and power generation.^17^ Rice husk is the most common agricultural waste, which makes up 25% of rice mass.^8^ About 66% of agricultural waste originates from cereal crop residues. Sugarcane waste, such as stems and leaves, is another major contributor to agricultural waste.^18^ Accordingly, agricultural residues are the preferred option for bioenergy generation owing to their abundant availability, low CO_2_ emissions, diverse products, and commercial potential.^21,22^ These residues have the potential to be recycled into valuable products instead of landfilling or incineration.^17^

### 2.2. Food processing waste

The food processing industry produces large amounts of wastes. These processes involve separation of the nutritional portion of raw fruits and vegetables from inedible parts.^23^ Approximately 38% of food waste is attributed to the food processing industry, where the main contributors to food waste are oil-bearing crops, fruits, and vegetables. As reported by the Food and Agriculture Organization (FAO) of the United Nations, about one third of the fruits and vegetables produced annually is converted into by-products in the food processing industry.^24,25^ The residues from processing fruits, vegetables, cereals, and oil crops include peels, seeds, shells, stems, and pulp.^26^

### 2.3. Energy crops

Energy crops are plants that are grown especially for energy generation, which can be utilized to produce biogas or liquid fuel.^27^ Oils in the form of fatty acids and lipids are stored in a variety of energy crop seeds, which can be converted to biodiesel and biofuel.^5^ The crops selected for bioenergy production should have the characteristics such as rapid growth, low cost, ability to adapt to severe soil and weather conditions, capability to offer high yields, low nutrient requirements, and not competing with the food production system.^7,28^ Examples of energy crops include cardoon, kenaf, sorghum, safflower, poplars, hemp, and rapeseed.^7^

### 2.4. Forestry waste

Forestry waste is generated from forests due to logging operations and wood industrial activities such as processing of timber, plywood, and pulpwood. Wood processing activities produce residues comprised of sawdust, barks, branches, split wood, *etc.*.^29^ In developed countries, wood processing residues are utilized for energy generation at the source of production to avoid wood waste transportation costs. The majority of energy produced from biomass utilizes wood and wood residues.^8^

### 2.5. Municipal solid waste

Municipal solid waste (MSW) is the waste disposed daily from households and from commercial activities, which can be organic and inorganic. Organic MSW includes paper, plastic bottles, food residues, textiles, wood, and furfural residues.^30,31^ Annually, around 1.3 to 1.9 billion tons of solid waste are generated globally, which is expected to reach 3.5 billion tons in 2050 due to the rapid increase in the human population and industrialization.^1,32^ MSW is typically disposed by landfilling, which could contaminate groundwater, in addition to the requirement of large land area. Nevertheless, the methane produced from landfilling can be used as an energy source.^8^ Thermal treatment by incineration is another common practice to reduce the solid waste volume and generate heat. However, it poses a threat to the environment due to the emission of pollutants such as nitrogen oxide, sulfur oxide, and fly ash.^32^

## 3. Biomass composition

**Fig. 2:**
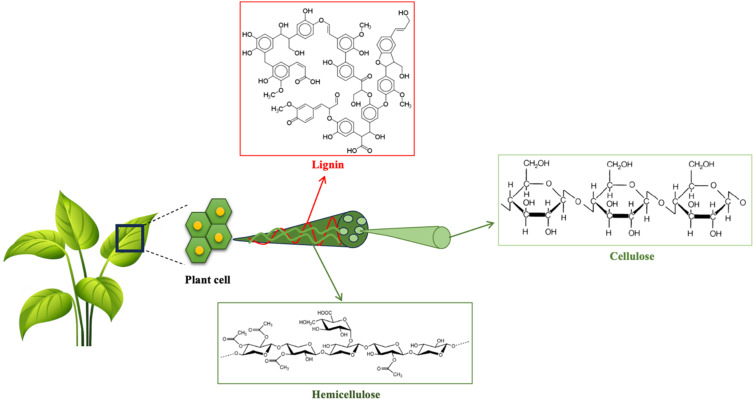
Lignocellulosic biomass components.

Lignocellulosic biomass is comprised of hemicellulose (15–30%), cellulose (40–60%), and lignin (15–30%), as shown in Fig. 2. The content of these three components varies depending on the type of biomass.^3^ In addition to the above-mentioned three constituents, biomass contains inorganic ash and minor fractions of extractives such as lipids, sugars, proteins, and starch.^33^ In plant cells, cellulose forms rigid microfibers, which function as the skeleton of the cells, whereas lignin and hemicellulose pack the inner cell space.^3^ Cellulose is a linear polysaccharide composed of long chains of glucose units linked by β-1,4-glysodic bonds, which tend to decompose when exposed to high temperatures. The abundance of OH groups in cellulose leads to extensive hydrogen bonding, involving both intra-chain and inter-chain interactions. Intra-chain hydrogen bonds stabilize the glycosidic linkages, while inter-chain hydrogen bonds promote the parallel stacking of cellulose chains.^34^ Cellulose rapidly degrades during fast pyrolysis as its weak β-1,4-glycosidic bonds cleave under acid or high-temperature conditions, reducing the degree of polymerization and producing furans and levoglucosan.^3^ Hemicellulose is a heterogeneous polysaccharide consisting of short pyranose and furanose sugar units such as d-xylose, d-mannose, d-glucose, d-galactose, l-arabinose, galacturonic acid, and glucuronic acid. It has a lower degree of polymerization compared to cellulose. Hemicellulose accounts for approximately 15–35% of the dry mass of plants, and its composition varies with plant species.^35^ The branched structure of hemicellulose accounts for its lower thermal stability compared to cellulose.^36^ Lignin is a branched polymer primarily composed of three aromatic basic units, *i.e.*, *p*-coumaryl (H), coniferyl (G), and sinapyl (S) alcohols. These three basic units mainly differ by the number of methoxy groups on their aromatic ring, and their H/G/S proportions largely depend on the biomass species. Lignin linkages are mainly ether bonds (60–70%), followed by carbon–carbon bonds (30–40%), while ester bonds are minimal and mostly found in herbaceous plants.^3,37^ The complex cross-linked aromatic network of lignin confers higher thermal stability than cellulose and hemicellulose.^3,36^

## 4. Thermochemical processes

**Fig. 3:**
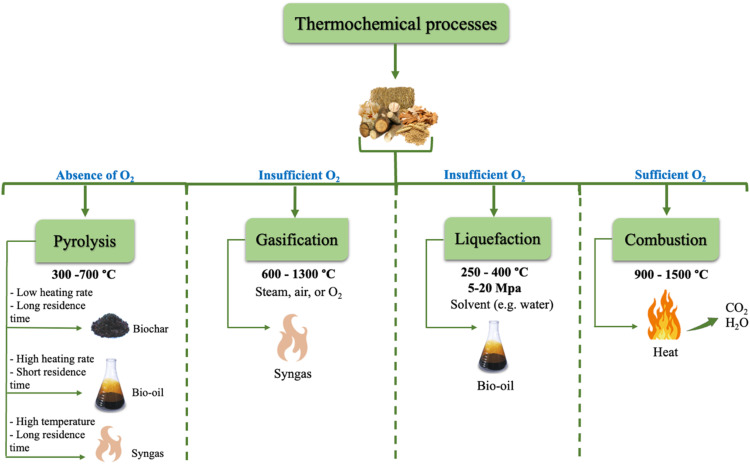
Various thermochemical processes and their products.

**Fig. 4:**
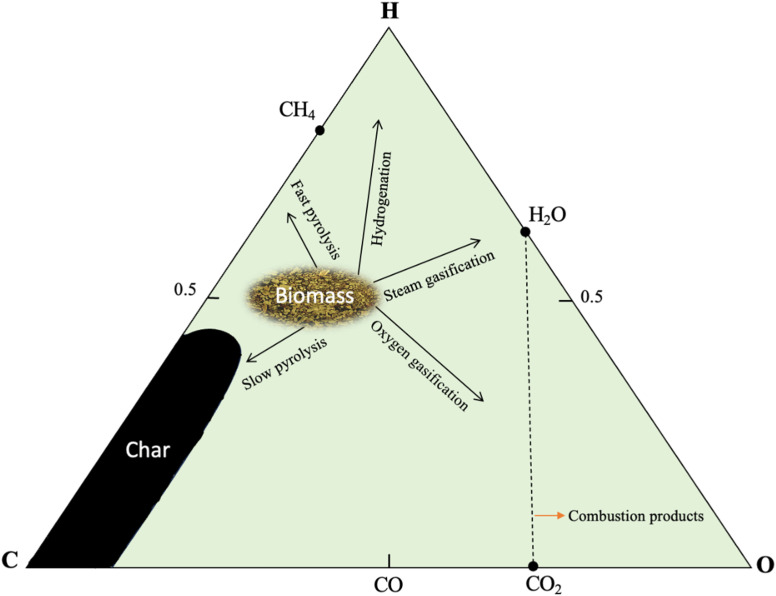
Ternary diagram of biomass thermochemical conversion, adapted from ref. 248 with permission from Elsevier, *Energy Convers. Manag.*, vol. 304, p. 118222, Copyright 2024.

The primary thermochemical processes are pyrolysis, gasification, liquefaction, and combustion,^11^ where all the aforementioned techniques involve the thermal degradation of the organic components in biomass.^1^ Thermal conversion treatment can recover the energy stored in biomass into heat in the case of combustion, or into biofuel *via* pyrolysis, gasification, and liquefaction.^37^ The choice of thermochemical technique is affected by several factors such as biomass feedstock type, desired end products, process economics, and environmental concerns.^1,2^Fig. 3 shows the four major thermochemical processes, their conditions, and target products. In the ternary diagram, as shown in Fig. 4, each corner represents 100% of a specific element (carbon, hydrogen, or oxygen). Char, being richer in carbon, is positioned at the carbon corner. For instance, biomass with a higher hydrogen and carbon content, is located near the carbon-hydrogen side in the diagram. Thermochemical processes decompose biomass components, shifting their positions in the diagram depending on the feedstock composition and the process applied. If biomass undergoes carbonization, its composition shifts toward the carbon corner of the ternary diagram. Conversely, hydrogenation enriches the product in hydrogen, moving it closer to the hydrogen corner.^38^

### 4.1. Pyrolysis

**Fig. 5:**
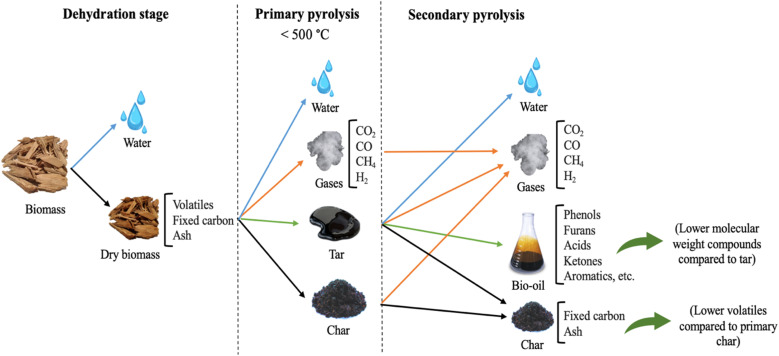
Different stages of pyrolysis.

Pyrolysis is a thermochemical process, which involves the irreversible decomposition of biomass due to the intense heat applied (300–700 °C) in the absence of oxygen to yield non-condensable gases, bio-oil, and char. The obtained products are affected by the process temperature, heating rate, residence time, and biomass nature. There are three main categories of pyrolysis, namely, fast, intermediate, and slow, depending on the residence time and the temperature. Fast pyrolysis has a short residence time and moderate temperature between 450–600 °C. It requires high heat transfer rates, which are attained by grounding the feed biomass. Fast pyrolysis mainly promotes the yield of liquid products (50–70 wt%).^5,37,39,40^ Alternatively, slow pyrolysis (carbonization) has a longer residence time and lower temperature (400 °C), and it produces approximately equal proportions of gases, bio-oil, and char.^37^ Pyrolysis occurs in three main stages, as shown in Fig. 5, where the first stage is for moisture removal. Further, the dry biomass undergoes primary pyrolysis, during which bond scission of its lignocellulosic components (hemicellulose, cellulose, and lignin) occurs, producing gases (CO, CO_2_, H_2_ and methane), H_2_O, tar, and primary char. At higher temperatures the primary products undergo further reactions such as dehydration, cracking, polymerization, and gasification, producing secondary products which include gases, bio-oil (phenols, ketones, aldehydes, furans, acids, aromatics, *etc.*), and secondary char.^40–42^ Both primary and secondary reactions can occur simultaneously. The produced char catalyzes the cracking of the primary products into lighter gases. Moreover, char gasification leads to the formation of gaseous products, and the polymerization reaction produces secondary char.^40,42^ The evolved gaseous products yield increases as the temperature increases, which is attributed to the degradation of primary char and tar. In contrast, the char yield decreases as the temperature increases due to the release of more volatiles. The maximum bio-oil yield is attained at approximately 450–500 °C.^43–46^ The pyrolysis of a diverse range of biomass materials has been reported in the literature, as listed in Table 1.

**Table 1:** Studies on the pyrolysis of biomass

Biomass	Reactor	Catalyst	Study purpose	Main findings	References
Hyacinth biomass (agricultural waste)	Solar pyrolysis reactor	No catalyst	To explore the effect of solar-torrefied biomass samples in improving the solar pyrolysis performance and product quality	-Higher torrefaction temperatures increased char (27.4 to 59.4 wt%), while reducing bio-oil (40.2 to 15.3 wt%) and syngas (32.4 to 15.3 wt%) yields	47
				-FTIR and GC-MS analyses showed that torrefaction reduces bio-oil acidity, lowering the corrosion potential	
Cotton stalk (agricultural waste)	Fixed-bed reactor	No catalyst	To investigate how reaction conditions influence the bio-oil yield from slow pyrolysis of cotton stalk	Bio-oil yield peaked at 38.5% at 500 °C with 0.5–1 mm cotton stalk particles, dominated by phenols (>40%). Under the same conditions, high-quality biochar was obtained	48
Orange peel, potato peel, pineapple peel, and corn cob (food processing waste)	TGA	No catalyst	To evaluate the potential of food processing waste-derived bio-oil as a source of biofuel and valuable chemicals	Producing transportation fuel fractions requires secondary catalytic treatment to reduce N/O-compounds and enhance pyrolytic oil quality	10
				-The produced bio-oil can be utilized as a source of chemicals (*e.g.* phenols and furans) that have pharmaceutical applications	
Sugarcane (agricultural waste)	Fixed bed batch reactor	No catalyst	To investigate the effect of different operating conditions on sugarcane slow pyrolysis to improve the yields of bio-oil, biochar, and evolved gases	-The highest bio-oil yield (44.7%) was obtained at 550 °C for a 16 cm height bed at 25 °C min^−1^ heating rate	49
				-The obtained bio-oil requires upgrading to be utilized as a fuel	
Oil palm mesocarp fiber and palm frond (agricultural waste)	Fixed bed reactor	No catalyst	To explore how the temperature affects the pyrolysis products formation	-The maximum bio-oil yield was obtained at 550 and 600 °C	46
				-The obtained bio-oil contained oxygenated and aromatic compounds	
Hornbeam shell residues (forest waste)	Fixed bed reactor	No catalyst	Optimizing pyrolysis conditions for enhanced bio-oil and biochar production	-The maximum bio-oil yield of 24.67% was reached at 550 °C, and the highest bio-char yield of 40.3% was attained at 400 °C	45
				-FTIR analysis revealed the dominance of oxygenated compounds	
Bamboo (agricultural waste)	TGA and laboratory-scale pyrolysis reactor	No catalyst	To study how the temperature affects slow pyrolysis behavior and the products yield	-The primary product was biochar	50
				-The maximum yield of bio-oil was 36.57% obtained at 500 °C	
Coconut shell, rice husk, sugarcane bagasse, cotton stalk, wheat straw and palm kernel shell. In addition to cellulose, hemicellulose, and lignin (agricultural waste)	Two stages fixed bed reactor	10 wt% nickel-based alumina catalyst	Exploring hydrogen and syngas production from different biomass samples utilizing two-stage pyrolysis-catalytic steam reforming	-Introducing steam and catalyst into the pyrolysis process enhanced hydrogen generation	51
				-The highest hydrogen yield obtained for palm kernels, and the lowest for wheat straw	
*Miscanthus* (agricultural waste)	Fluidized bed system for pyrolysis, and fixed bed reactor for bio-oil upgrading	Pd/C	To perform qualitative and quantitative analyses of the obtained and upgraded bio-oil	-The oxygenated and sulfur compounds were reduced in the upgraded bio-oil, and the heating value was increased	52
				-The upgraded bio-oil is a suitable substitute for transportation fuel	
Wood and wood barks of *Ficus religiosa* (forest waste)	TGA and laboratory-scale fluidized bed reactor	No catalyst	To investigate the pyrolysis behavior of the feedstock under various operating conditions, in addition to conduct complete product distribution *via* FTIR-GC/MS analysis	-The highest yield for bio-oil of 47.5% obtained at 450 °C	53
				-The obtained bio-oil comprised a variety of organics that can be utilized in the chemical industry	
Orange pulp waste (food processing waste)	TGA	No catalyst	To provide a detailed analysis for the thermal degradation behavior, kinetics, and evolved gas analysis	-Activation energy calculation revealed the complexity of the reactions	54
				-The main products are CO, CO_2_, and H_2_O, in addition to oxygenated, aliphatic, and aromatic compounds	
Rapeseed (energy crop)	TGA	No catalyst	To examine the influence of heating rate on rapeseed slow pyrolysis	As the heating rate increased, the maximum DTG curve peak temperature decreased	55

The co-pyrolysis of lignocellulosic biomass and synthetic polymers is an efficient pathway for upgrading bio-oil, owing to the synergistic effect.^56^ Plastic has a high H/C ratio and low oxygen content, which support the low H/C and high O/C ratios in biomass, respectively. During co-pyrolysis, plastic acts as a hydrogen donor, which improves the quality and the heating value of the obtained bio-oil.^57,58^Table 2 lists the studies related to the co-pyrolysis of plastic and biomass.

**Table 2:** Studies on biomass and plastic co-pyrolysis

Biomass/plastic blend	Reactor	Purpose of the study	Main findings	References
Lignin/PP, HDPE and LDPE	TGA	To explore the potential valorization of these materials *via* co-pyrolysis	Py-GC/MS revealed that lignin-LDPE/HDPE mixtures at 600 °C and a 1 : 3 ratio achieved the highest compound recovery	59
Almond shell/HDPE	TGA	To explore the synergistic influence on the pyrolytic oil yield, and to investigate its potential as a bioenergy source	Co-pyrolysis reduced oxygenated compounds and enhanced aliphatic formation	60
Pine woodchips/waste-tyre scrap	Fixed bed reactor and auger reactor pilot plant	To explore the co-pyrolysis of pine woodchips and waste tyre scraps to upgrade the bio-oil quality	The acidity, density and oxygen content for the obtained bio-oil decreased	61
Oil palm trunk (OPT)/PP	TGA	To study the characteristics of the bio-oil obtained from OPT pyrolysis and co-pyrolysis of OPT and PP	PP presence improved hydrocarbons production and decreased the contents of phenolics in the co-pyrolysis oil	62
Grape stems and plastic waste (PE, PP, and PS)	TGA	To explore the thermal behavior, pyrolytic characteristics, and the synergistic effect of the blend co-pyrolysis	-At the early stages, softened plastic enveloped the biomass and hindered the release of volatiles	63
			-Intermediates generated from biomass breakdown enhanced plastic chain degradation	
			-Hydrogen radicals released from the plastic enhanced the formation of aromatics	
Lignin/plastics (PE, PP, PS, and PC)	Semi-batch glass reactor	To determine the yields and the composition of the products	-The degradation of lignin was reduced, whereas that of plastic enhanced	64
			-PC/lignin blend produced the highest phenols	
Pine wood sawdust, bamboo, and empty fruit bunch/HDPE and PS	TGA	To investigate and compare the thermal degradation of different plastic and biomass blends	-The degradation of the individual biomass showed one single peak in the DTG curve, while that of the blends showed two peaks	65
Cotton stalk, hazelnut shell, sunflower residue, and arid land plant *Euphorbia rigida*/PCV and PET	TGA	Comparison between the thermal degradation behavior of biomass and its blend with plastic	-The thermal behavior of biomass and plastic was different owing to their structural differences	66
			-Higher activation energies needed for plastic degradation compared to biomass	

**Fig. 6:**
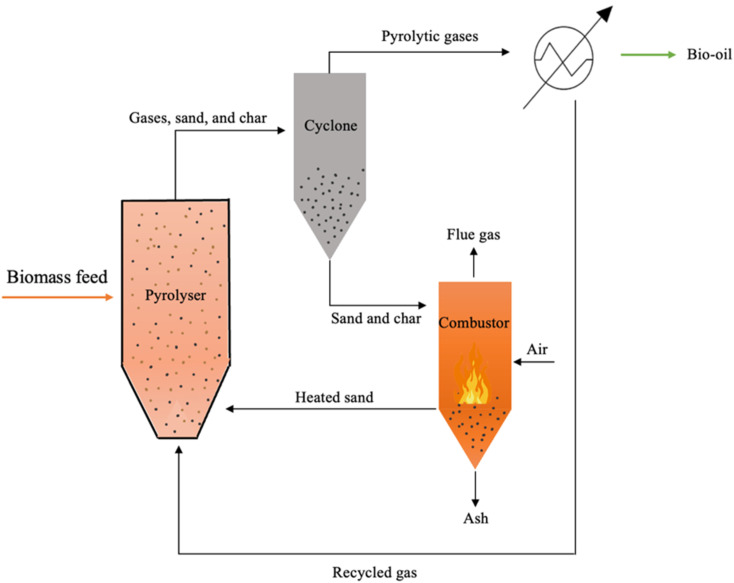
Schematic of a circulating fluidized bed reactor (CFBR).

**Fig. 7:**
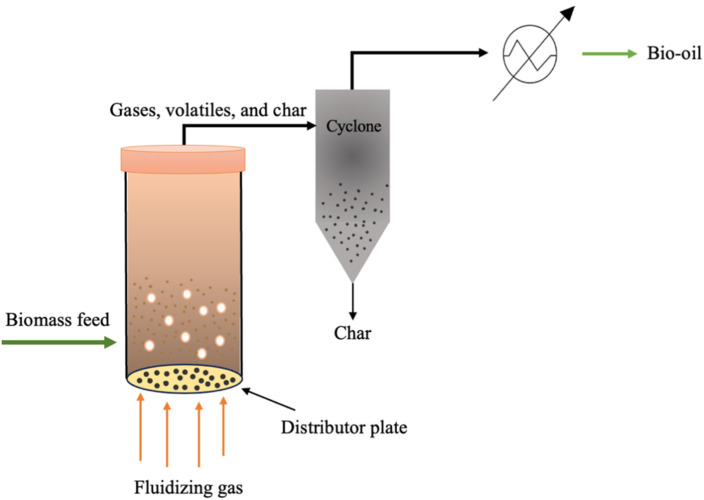
Schematic of a bubbling fluidized bed reactor (BFBR).

**Fig. 8:**
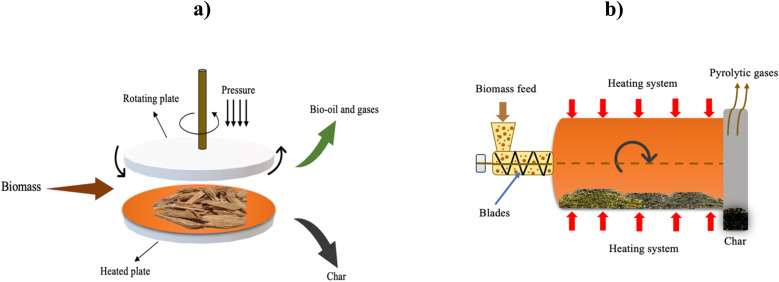
Schematic of (a) ablative pyrolysis reactor and (b) rotary kiln reactor.

**Fig. 9:**
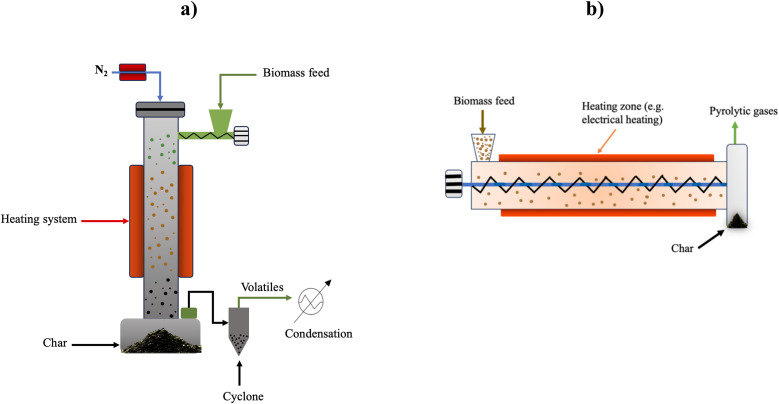
Schematic of (a) entrained flow reactor (EFR) and (b) Auger reactor.

Several pyrolysis reactors have been utilized for the pyrolysis of biomass such as fluidized bed reactors (FBRs), in which biomass particles are mixed with sand and heated rapidly. The bed is heated by combusting a portion of the produced gas and char. Sand is utilized as an inert bed solid, which provides heat transfer to the biomass feed.^67^ Fluidized bed reactors are classified into three types, which are circulating fluidized bed (CFBR), bubbling (BFBR), and entrained reactors (EFBR), as shown in Fig. 6, 7, and 9a, respectively.^68,69^ Fluidized bed reactors are easy to scale up, have a simple design, and can be utilized for fast pyrolysis due to the ease of controlling the residence time by controlling the fluidized gas flow rate. However, FBRs are large and require high construction and operational costs.^39,70^ CFBRs consist of two interconnected units, one for pyrolysis and one for char combustion, enabling continuous circulation of heated sand. Operating at high gas velocities, they ensure short biomass residence times, effective gas–solid contact, uniform mixing, and improved temperature control. Similarly, BFBRs use an inert gas and sand to fluidize small biomass particles, ensuring high heating rates, uniform temperatures, and efficient continuous operation with high bio-oil yields; however, they require careful control of heat transfer and are sensitive to the biomass type, particle size, and gas velocity. In EFBRs, dried biomass is fed at a controlled rate and carried into the reactor by preheated gas. The particle velocity is determined by the gas flow, and pyrolysis occurs as the biomass passes through the heated reaction zone.^68,69^ Auger (screw) reactors (Fig. 9b) are tubular, continuous systems where biomass is conveyed by a rotating screw, while heat is supplied through the reactor walls. The screw enhances mixing and contacts with the heated surface, promoting efficient pyrolysis. A key advantage of this design is its compactness and potential portability, enabling on-site biomass conversion, which lowers operational costs by reducing feedstock transportation to biorefineries.^39,71^ Ablative reactors are another type of pyrolysis reactor in which the biomass particles are subjected to a pressure against a heated plate, causing biomass thermal erosion at the hot contact layer, as depicted in Fig. 8a. This type of reactor is capable of processing the large particle size of biomass, and its efficiency depends on the applied pressure and the plate surface temperature. Ablative pyrolysis is influenced by the heated plate surface area, thus scaling up could be challenging.^72^ Rotary kiln reactors involve a continuous slow pyrolysis process, in which biomass move by blades installed in the inner rotary tube, as shown in Fig. 8b. The biomass is heated by the flue gas produced from syngas combustion. This technology has high efficiency and stable product formation, and it is the most utilized reactor for municipal solid waste pyrolysis. Rotary kiln pyrolysis technology is applied in China for heating purposes in rural areas.^73^ Other examples of typical pyrolysis reactors include rotating cone and vertical kiln.^39,73^

Over the past, significant efforts have been devoted to commercializing the pyrolysis process. However, although the reaction mechanisms have been extensively studied, other aspects, such as reactor control systems, still require further research. Biomass pyrolysis faces challenges in sustaining continuous operation and producing high-quality bio-oil. Although catalysts and hydrotreatment have been used to improve the quality, rapid catalyst deactivation and high costs have hindered its commercialization. In this case, several companies are pursuing commercialization, and a number of moderate-scale demonstration and commercial plants has already been established.^74,75^Table 3 lists some of the commercial pyrolysis plants.

**Table 3:** Commercial biomass pyrolysis units located worldwide^76,77^^.^^a^

Country	Reactor	Biomass	Capacity	Status
Brazil	CFBR	Forest residues	83 ML year^−1^	Under development (in 2023)
Canada	CFBR	Wood residues	38 ML/year	Active since 2018
Canada	CFBR	Mill and forest residues	11 ML year^−1^	Active since 2006
Canada	Auger reactor	Wood residues	6 ML year^−1^	Under development (in 2023)
Finland	Rotating cone	Sawdust and wood residue	20 ML year^−1^	Active since 2020
France	—	Cellulosic biomass	0.8 ML year^−1^	Under development (in 2023)
The Netherlands	Rotating cone	Wood biomass	20 ML year^−1^	Active since 2015
Sweden	Rotating cone	Sawdust	21 ML year^−1^	Active since 2021

a ML: million liters.

### 4.2. Gasification

**Fig. 10:**
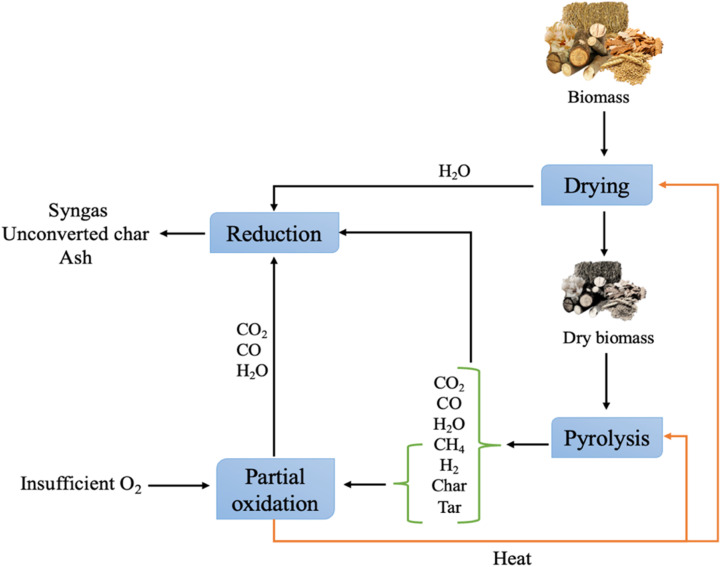
Main reactions in the gasification of biomass.

**Fig. 11:**
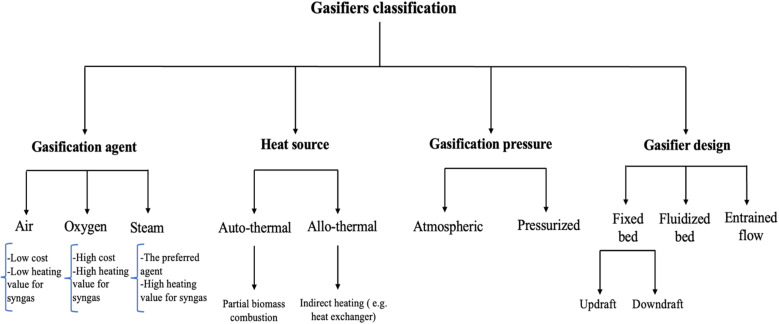
Classification of gasifier reactors.

**Fig. 12:**
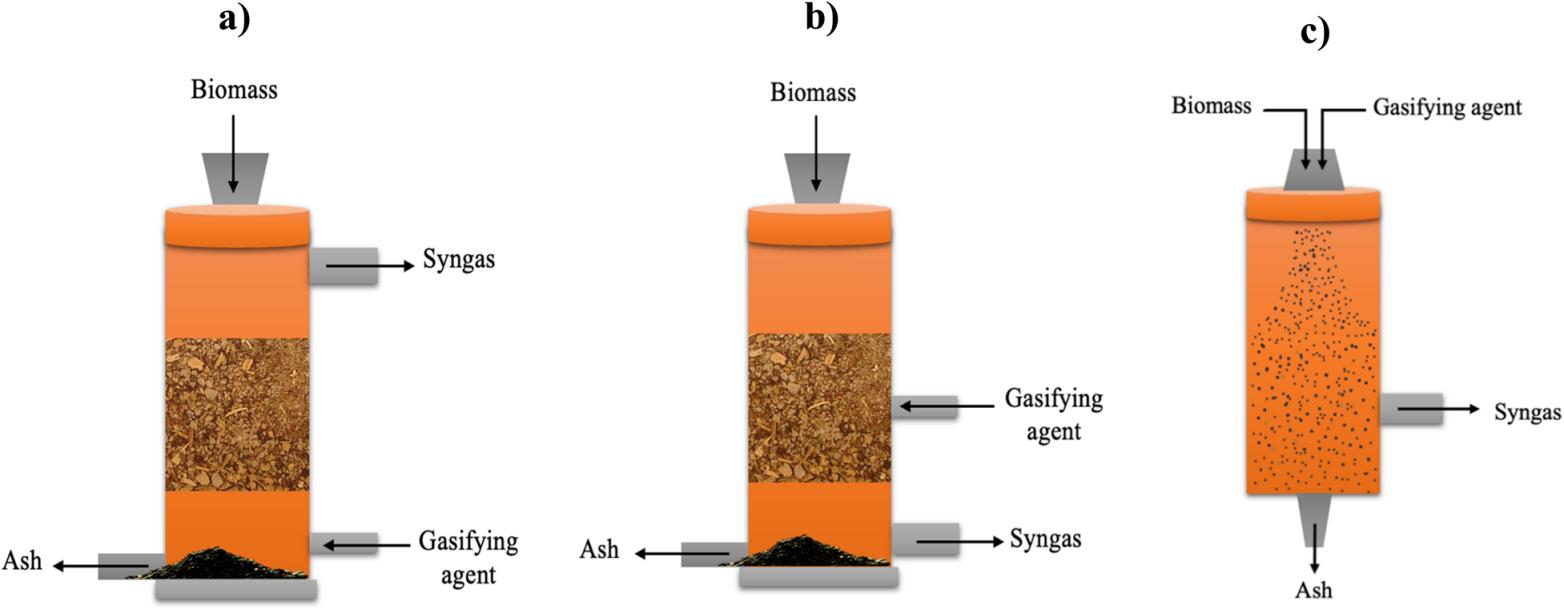
Schematic of (a) updraft gasifier, (b) downdraft gasifier, and (c) entrained flow gasifier.

The gasification process involves converting solid and liquid biomass into gaseous products, usually syngas, along with unconverted char and ash, by applying high temperatures.^8,78,79^ This is a crucial process for green hydrogen production, which can be applied in fuel cell vehicles, power generation, and industrial processes, helping to reduce emissions.^80^ The gasification process occurs in the presence of gasifying agents, which can be air, oxygen, steam, and their combination, where each agent yields different compositions and heating values for the obtained syngas. Gasifying agents react with carbon and heavy hydrocarbon, producing lower molecular weight gases.^81^ Heat is provided either through partial combustion of the products (auto-thermal) or from an external source (allo-thermal). As depicted in Fig. 10, the gasifying process involves four main reactions, including drying (below 150 °C), pyrolysis (150–700 °C), oxidation (700–1500 °C), and reduction (800–1100 °C).^82^ In the pyrolysis reaction, as discussed earlier, biomass decomposes, releasing gaseous products, tar, and char. The pyrolysis products undergo partial oxidation, producing CO, CO_2_, and water, while also releasing the thermal energy needed for other endothermic reactions (drying and pyrolysis). Reduction reactions incorporate all the pyrolysis products to yield the final syngas.^79,83^ The properties of the products depend on the biomass feed type, gasifying agent, catalyst, and temperature.^83^ Gasifier reactors are classified according to the gasifying agents, heat source, and gasifier pressure and design, as shown in Fig. 11. Among them, fixed bed gasifiers are the simplest in design, which consist of a bed of solid fuel particles in which the gasifying agent moves through. They are classified into updraft and downdraft reactors. In updraft gasifiers, the gasifying agent and syngas move up (Fig. 12a), whereas in downdraft gasifiers, the syngas moves down (Fig. 12b). Fixed bed gasifiers can handle biomass with high humidity and different sizes and have a high solid residence time, high thermal efficiency, and high carbon conversion. However, fixed bed gasifiers require the treated feed to be homogenous and have the same characteristics.^79,84,85^ Entrained flow gasifiers are another type of reactor, in which the gasifying agent and biomass are fed in co-current, as shown in Fig. 12c. This type of gasifier operates at high temperature (1200–1500 °C) and requires fine feedstock particles for more efficient heat transfer.^79,81,86^ Usually, torrefaction pre-treatment of the feedstock is required to reduce the moisture content and minimize the bulk density.^87,88^ Torrefaction is a thermochemical pretreatment used to upgrade raw solid biomass for gasification. Torrefaction involves three main reactions including depolymerization, devolatilization, and decomposition.^89^ This process, which improves fuel quality by removing moisture and breaking down rigid fibrous structures, depends on the reaction temperature and type of reaction medium. It is classified into dry (200–300 °C) and wet (180–260 °C). Wet torrefaction is suitable for wet biomass, while dry torrefaction is applied to solid biomass under either oxidative or non-oxidative conditions. Dry torrefaction is commonly used to improve biomass by increasing its energy density and reducing its moisture, oxygen, and volatile content.^90,91^ In many cases, torrefaction is combined with pelletization to increase the bulk density of the material.^79^ Abdulyekeen *et al.*^92^ evaluated torrefied wood and garden waste in a helical screw fluidized bed reactor at 250–300 °C by analyzing their grindability, thermal, and structural properties. The results showed that temperature had the greatest impact, with higher temperatures reducing the volatile matter, oxygen, and hydrogen, while increasing the fixed carbon, calorific value, and carbon content. Another recent study by Silveira *et al.*^93^ explored torrefaction (225–275 °C, 60 min) to increase the energy density of a six-species waste wood blend. TGA was used to examine the combustion behavior, and the emissions were numerically assessed. Torrefaction reduced the H/C ratio from 1.87 to 1.05 and the O/C ratio from 0.70 to 0.47, improving the thermal stability and altering the ignition dynamics, thereby enhancing the overall efficiency of the biofuel. Table 4 lists the studies pertaining to the gasification of various biomass materials, and Table 5 presents some of the industrial applications of biomass gasification.

**Table 4:** Studies related to biomass gasification

Biomass	Gasifier type	Study purpose	Main findings	References
Oil palm wastes (oil palm fronds, empty fruit bunches, oil palm trunks, and palm kernel shells) (agricultural waste)	Aspen plus simulation	To explore hydrogen production from four oil palm wastes *via* biomass gasification, in addition to techno-economic, and environmental analysis	-The highest hydrogen yield was obtained from oil palm trunks	80
			-Hydrogen production from oil palm waste gasification has a longer payback period but lower environmental impact than conventional steam methane reforming	
Food waste	Batch reactor	To explore the potential of supercritical water gasification technology for energy generation and the feasibility of hydrogen production in Bangladesh	Electricity generation from food waste in Bangladesh could increase from roughly 489 MW in 2023 to about 2042 MW by 2042	94
Coconut waste (food processing waste)	Gasification reactor	To explore how NaCl concentration, reaction time, and temperature affect hydrogen production from coconut oil meal gasification	Higher gas yields and H_2_ selectivity were achieved at 550 °C, 75 min reaction time, and with 10% NaCl addition	95
Oak and corn stalk (agricultural waste)	Fixed-bed reactor	To analyze the syngas, tar, and biochar generated from oak and corn stalks under varying temperatures using electric gasification and microwave gasification	-Microwave gasification produced 3–7% more syngas than electric gasification	96
			-Microwave gasification yielded 3–6% more biochar than electric gasification, while electric gasification generated more tar in the 600–800 °C range	
Wood	Solar furnace	To explore the potential of syngas generation from wood utilizing prototype solar reactor	The prototype solar reactor was reliable for the gasification of both biomass and waste	97
Rice husk pellet (agricultural waste)	Bubbling fluidized bed	To explore the advantages of combining torrefaction and gasification processes	Torrefaction process contributed in increasing the heating value of rice husk pellet. Conversely, the electric energy demand for the process reduced the process efficiency	98
Pine sawdust (forest waste)	Fluidized bed gasifier	To explore the feasibility of syngas generation from catalytic gasification of biomass	High H_2_/CO syngas was obtained which can be utilized in methanol production	99
Cellulose as a model compound	Fluidized bed gasifier	To investigate cellulose gasification utilizing Rh/CeO_2_/SiO_2_ catalyst in a fluidized-bed reactor at low temperature	Utilizing Rh/CeO_2_/SiO_2_ as a catalyst introduced a novel approach for hydrogen and syngas generation at low temperature	100
Municipal sludge	Downdraft fixed bed gasifier	To explore the municipal sludge gasification based on the sludge characteristics, and to study the produced solid residues properties	-The high volatile organics in the sludge indicated that the sludge can be valorized *via* gasification	101
			-Ash content was higher than the common biomass	
			-The organic functional groups determined by the FTIR analysis in the char were quite similar with those in sludge	
Manure (animal waste), sewage sludge and date pits (food processing waste)	Fixed bed gasifier	To study the effect of pressure, temperature, and ratio of oxygen on the composition and the heating value for the syngas	-The properties of the obtained syngas can be controlled by optimizing the biomass feedstock mixing	102
			-Changing the operating conditions affected the syngas composition and LHV	
Pine sawdust agricultural waste	Pyrolysis-gasification coupled reactor	To investigate the production of hydrogen-rich syngas utilizing a pyrolysis-gasification coupled reactor	-CO_2_ was the highest in the pyrolysis part, whereas H_2_ was the highest in gasification part	103
			-The main carbon conversion occurred in the pyrolysis part	
			-The coupled reactor produced less tar	
Wood, paper, municipal solid waste, and sawdust (forest waste and MSW)	Downdraft gasifier	Using different gasification agents (air, oxygen, oxygen-enriched air, and steam) and comparing the obtained products	-The highest hydrogen production was associated with steam. Conversely, the lowest was associated with air	104
			-Increasing the gasification temperature decreased hydrogen production and reduced the syngas calorific value for all the four agents	
			-Increasing the biomass feeding rate adversely affected the hydrogen production and energy and exergy efficiencies	
Pine sawdust and HDPE blend. Agricultural waste and plastic	Fixed bed reactor	To explore the efficiency of Ni-Fe@nanofibers/porous carbon catalyst for syngas generation through pyrolysis-gasification process for the blend	The optimum biomass-plastic ratio was 1/2, and the best syngas quality was obtained at a gasification temperature of 700 °C	105

**Table 5:** Industrial application of biomass gasification^78,106^

Country	Reactor type	Biomass type	Product
Austria	—	Wood chips	2 MW electrical power and 4 MW heat
Denmark	Circulating fluidized bed (CFB)	—	6 MW electrical power and 12 MW heat
Finland	Entrained flow gasifier (EF)	Biomass and sludges, RDF	10–200 MW heat
Canada (Toronto MSW plant)	—	Municipal solid waste	Electricity
Netherlands (Americentrale fuel gas plant)	—	Demolition wood	Fuel gas
Germany (Fendoteoc gasification plant)	—	Municipal solid waste	Electricity

### 4.3. Liquefaction

**Fig. 13:**
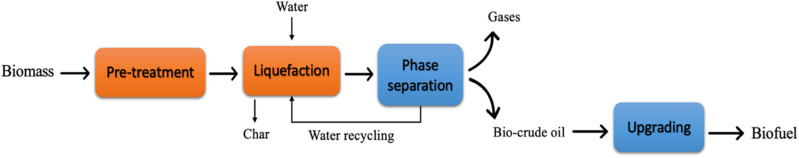
Typical biomass hydrothermal liquefaction process.

Liquefaction is another thermochemical process that aims to produce bio-oil, in addition to fractions of gaseous product and solid residues by applying a relatively low temperature (250–400 °C) and high pressure (5–20 MPa).^107,108^ The major difference between liquefaction and other thermochemical techniques (pyrolysis and gasification) is the utilization of solvents such as methanol, ethanol, and water as the reaction medium during the liquefaction process.^108^ Organic solvents act as hydrogen donors, which improve the bio-oil yield with a low oxygen content. Hydrothermal liquefaction (HTL) takes place under hot pressurized water conditions. The HTL process is comprised of three main steps; firstly, depolymerization of the macromolecules into shorter chains. Subsequently, dehydration and decarboxylation reactions convert the oxygen into water and CO_2_. The last step involves repolymerization of the small monomers to form coke, which occurs in the case of hydrogen deficiency.^5,109,110^ The formed aqueous phase can be recycled to the liquefaction reactor, which will reduce the water demand, as shown in Fig. 13, whereas bio-crude oil will require upgrading for further uses.^5,109^ The high solvent cost and the environmental concerns related to solvent recycling are the main disadvantages of utilizing organic solvents.^108,111^ The liquefaction process can handle wet feedstock, which eliminates the requirement of the feed drying process in the case of using water as the solvent.^108,112^ The liquefaction process is affected by chemical factors including biomass type and size, catalyst, solvent, and physical factors such as pressure, temperature, heating rate, and residence time.^107^ Homogenous catalysts (*e.g.* organic and inorganic acids) are typically used to reduce char formation.^107^ Moreira-Mendoza *et al.*^113^ studied HTL of agro-waste, microalgae, and defatted seeds, finding that lipid-rich feedstocks gave higher biocrude yields (∼65%) and energy content, while lignocellulosic biomass produced only 10–20%. Using Ni-Pt/Al_2_O_3_ or Pd-C/Al_2_O_3_ with glycerol enhanced deoxygenation and improved the biocrude quality. Another recent study by Awadakkam *et al.*^114^ investigated HTL of Canadian spruce and poplar, identifying them as promising feedstocks. Using an ethanol–water co-solvent with K_2_CO_3_ improved the biocrude yield, reduced the oxygen content, and increased the energy density. The co-solvent also acted as an *in situ* hydrogen donor, minimizing the external hydrogen demand and enhancing the process efficiency. A similar study by Ocampo *et al.*^115^ investigated HTL of microalgal biomass using a water-acetone mixture, achieving ∼60 wt% biocrude yield at 5 wt% acetone. This process is economically favorable but produces biocrude with high nitrogen (23.68%) and oxygen (10.68%) contents, requiring further refining.

**Fig. 14:**
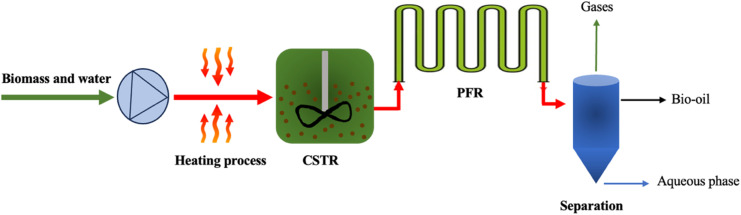
CSTR-PFR configuration in HTL, adapted from ref. 116 with permission from Elsevier, *Energy Convers. Manag.*, vol. 302, p. 118093, Copyright 2024.

Most HTL research has been carried out in batch reactors, which are simple, robust, and capable of operating under a range of reaction conditions. However, although they are versatile for experimentation, batch reactors are unsuitable for industrial-scale biofuel production. Batch HTL operates intermittently, limiting the efficiency and scalability, whereas continuous HTL offers a more efficient and controllable pathway for bio-oil production.^116^ A continuous stirred tank reactor (CSTR) uses an impeller and electric heater to ensure thorough mixing and maintain the desired reaction temperature, respectively, enhancing the heat transfer compared to a plug flow reactor (PFR). Although a CSTR alone provides a lower biomass to bio-oil conversion due to its shorter residence times, combining it in series with a PFR extends the residence time, increases the bio-oil yield, and minimizes plugging issues with lignocellulosic feedstocks.^116–118^ This CSTR-PFR configuration, as depicted in Fig. 14, demonstrates strong potential for HTL processes.^117^Table 6 lists the studies related to biomass liquefaction.

**Table 6:** Studies related to biomass liquefaction

Biomass	Solvent	Study purpose	Main findings	References
Softwood trees residues (forest waste)	Water	To investigate a novel method for biofuel production by combining HTL with pipeline hydro-transport of biomass	-The biocrude has a low oxygen and water content and high heating value, but high acidity and carbon residue indicate the need for catalytic upgrading	119
			-Integrating pipeline hydro-transport with HTL is technically feasible for biofuel production, supporting its future commercial implementation	
Oil processing residues, agricultural residues, forest residues, and food waste	Water	To examine biocrude production from different feedstocks *via* HTL, highlighting how biomass composition influences the HTL process	-Mustard meal produced the highest biocrude yield, followed by canola meal, under the optimized conditions of 320 °C, 30 min residence time, and a 5 : 1 water-to-biomass ratio (wt/wt)	120
			-The optimum 1 : 3 canola to mustard meal ratio yielded 41.9 wt% biocrude with a low oxygen content	
Wood bark (forest waste)	Water	To compare the liquid composition produced *via* slow pyrolysis and HTL processes for different wood types	-HTL liquids contained mainly water, acetic acid and catechol	121
			-Slow pyrolysis liquids composed of a variety of phenolic compounds and furfurals	
			-Similar concentrations of aromatics and phenols were found for HTL and slow pyrolysis	
Bamboo (agricultural waste)	Phenol, ethylene glycol (EG), and ethylene carbonate (EC)	To explore the effect of different organic solvents, liquid ratio, and reaction time on liquefaction yield and products	-Phenol is the best solvent for bamboo liquefaction	122
			-Similar low boiling point products were identified by GC-MS, irrespective of the solvent type	
Green waste mixture of straw, wood, and grass (agricultural waste)	Tetralin	To investigate the products of green waste liquefaction using Raney Ni catalyst and tetralin as the solvent	-The catalyst enhanced methane formation	111
			-The catalyst lowered the oxygen content and increased the yield of *n*-hexane	
Pinewood (forest waste)	Water, ethanol and acetone	To investigate the effect of different solvents on the biomass liquefaction process	-Acetone as the solvent resulted in the highest conversion rate	123
			-The product distribution was strongly affected by the solvent type	
Silver birch (forest waste)	Water	To study the liquefaction of silver birch wood in liquid water using sodium carbonate as the catalyst and to analyze the products *via* FTIR and GC-MS	-53.3% heavy oil yield was obtained at a reaction temperature of 380 °C	124
			-FTIR and GC/MS analysis revealed the formation of aldehyde, ketone, hydroxybenzene and ester	
*Onopordum heteracanthum* stalks (agricultural waste)	Methanol, ethanol and acetone	To explore the liquefaction of *Onopordum heteracanthum* stalks with catalysts (KOH and ZnCl_2_) and without catalyst at three temperatures and to explore the potential of bio-oil production	-In non-catalytic liquefaction, the best operating conditions were with acetone at 563 K	125
			-ZnCl_2_ catalyst was found to be more effective than KOH	
*Cunninghamia lanceolata* (forest waste)	Water	To study the direct liquefaction of *Cunninghamia lanceolata*	The highest heavy oil yield occurred at 280–360 °C	126
*Pinus banksiana* (forest waste)	Ethanol	To investigate the liquefaction of Jack pine wood powder in H_2_ environment using sub/super-critical solution of ethanol and iron-based catalysts (FeS or FeSO_4_)	-Increasing the residence time, H_2_ pressure, and reaction temperature enhanced the oil yield	127
			-(FeS and FeSO_4_) catalysts improved the oil production	
			-GC-MS revealed that phenols were the dominant in the bio-oil	

The demonstration and commercial plants for HTL are listed in Table 7.^76,77^

**Table 7:** Biomass HTL plants^76,77^^a^

Country	Plant scale	Biomass	Capacity	Status
Canada	Commercial	Forestry waste	8 ML per year (bio-oil)	Under development (in 2023)
Australia	Demonstration	Post-consumer waste	1.6 ML per year (bio-oil)	Active since 2012
Canada	Demonstration	Sewage sludge	2 dry tonnes per day	Under development (in 2023)
Denmark	Demonstration	Wastewater sludge	5 ML per year (bio-oil)	Under development (in 2023)
India	Demonstration	Food waste, sludge, and algae	80 L per day (bio-oil)	Active since 2016
Norway	Demonstration	Forest waste	1.5 ML per year (bio-oil)	Active since 2021
Turkey	Demonstration	Different wastes	8.7 ML per year (bio-oil)	Active since 2011

a ML: million liters.

### 4.4. Combustion

The combustion process is comprised of three primary stages, *i.e.*, drying, evolution of volatiles, and char oxidation. The combustion of the released volatiles is the primary source of the generated heat, which is utilized to produce steam for electricity generation. Char is further oxidized by O_2_, CO_2_ and H_2_O and only ash remains.^37,128^ Biomass combustion is the most common thermochemical process due to is low cost and processing speed. However, the complexity of biomass and the differences in its chemical structure pose challenges in burner design and control of the operating conditions.^129^ Moreover, the high content of moisture and volatiles in biomass in comparison to coal complicates the combustion performance.^130^Table 8 summarizes the studies on biomass combustion and co-combustion with coal.

**Table 8:** Studies related to biomass combustion and co-combustion

Biomass type	Burner/rector type	Study purpose	Main findings	References
Rice straw and wheat straw	Fixed bed combustor	To study the combustion behavior of rice straw and wheat straw biomass under different operating conditions	It was found that a high air temperature reduced the time required for the volatiles and char to burn	131
Olive residue, pine sawdust and sugarcane bagasse	Drop tube furnace	To study biomass combustion under both oxy-fuel and air conditions and to compare with coal combustion characteristics	-Biomass flame was less sooty than that of coal	132
			-Changing from air to oxy-fuel hindered the combustion intensity	
Spruce bark, short rotation coppice (SRC) poplar, Danish straw, and torrefied wood	Single particle reactor (SPR)	To investigate the NO release during char combustion	NO reduced inside the char particles got released as the conversion progressed	133
Lignite, vine pruning, olive tree pruning, corn stalk, and poultry litters	TGA	To investigate the potential of agricultural and animal residues as fuels for co-combustion with lignite	-Torrefaction process enhanced the char reactivity	134
			-Mixing torrefied biomass with lignite decreased the ignition temperature compared to individual lignite	
Corncob, hardwood, and bituminous coal	TGA	To study the effect of heating rates, mixing ratios, and biomass type on combustion characteristics	-Biomass showed better combustion behavior than coal	135
			-Increasing the heating rates improved combustion performance	
Pine, eucalyptus, and willow	Natural gas burner	To find correlations between particle mass and the combustion behavior	Derivation of empirical formulas for the duration of each combustion stage	136
Palm kernel shell	Plug flow reactor	To investigate biomass combustion properties at elevated temperature and high heating rate that reflect the real furnace environment	-Char combustion required a higher residence time in comparison to drying and devolatilization stages	130
			-Particle size showed the major effect on char oxidation, while it was less impactful at drying stage	
Beech wood	Entrained-flow reactor	To formulate a model representing the combustion of woody biomass, and to assess its accuracy	The developed model can predict various parameters during biomass combustion	137
Naomaohu coal and pine sawdust	TGA	To investigate the mass decay behavior and the volatile products of biomass and coal and their blend combustion under inert and air conditions	-Blending pine dust with coal decreased the ignition time and NO emissions	138
			-Oxygen level in pine dust contributed to C=O functional group formation	
			-The co-combustion exhibited a synergistic behavior under air atmosphere	
Peanut shells and coal gangue	TGA	-To predict the co-combustion characteristics of coal gangue and peanut shell using ANN model and compare it with experimental results	-The activation energies of coal and peanut shell co-combustion were less than those for individual components	139
		-To investigate the thermodynamic, kinetic properties, and evolved gases *via* FTIR	-Combustion products identified by FTIR were H_2_O, CO_2_, CO, CH_4_, C=O, phenols, C–O and NH_3_	
			-ANN20 was the most accurate model to predict CG and PS co-combustion	

**Fig. 15:**
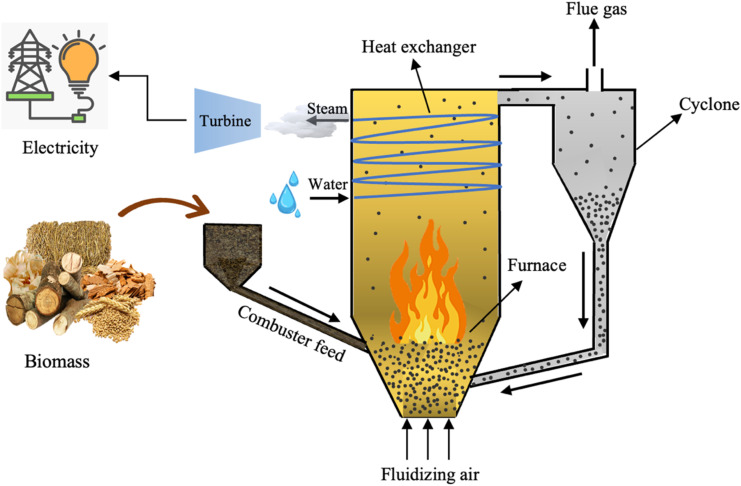
Schematic of a typical CFBC system.

In the past, biomass combustion was not considered an energy source for power plants owing to its low heating value compared to fossil fuels and its inconsistent composition.^140,141^ However, fossil fuel depletion and escalating costs, in addition to the emerging environmental concerns have led to an interest in utilizing biomass for energy generation *via* co-combustion with coal.^141^ Many existing large-scale power plants utilize biomass as a feedstock, as enlisted in Table 9. Fluidized bed combustors (FBC) are optimal technology for large-scale power generation plants, given that they have the ability to handle different feed sizes, moisture contents, shapes, feedstock pulverization is not required, also it have a uniform temperature distribution and high heat transfer coefficient between the bed and heat exchanger.^141–143^ However, there are some disadvantages such as the requirement of an efficient solid–gas separation system, boiler erosion, and agglomeration of the bed for some types of herbaceous biomass.^142^ A typical circulating fluidized bed combustion system (CFBC) is shown in Fig. 15.^142,144^

**Table 9:** Large-scale power plants utilizing biomass^141,145,146^

Power plant location	Biomass	Combustion system	Capacity (MW)
Power plant at Port Talbot in Wales/UK	Wood	CFB	350
Alholmens Kraft power plant/Finland	Pulp, paper and timber residues	CFB	265
Polaniec biomass power plant/Poland	Tree-farming and agricultural by-products	CFB	205
Power plant in Florida/USA	Sugar cane fibre (bagasse) and recycled urban wood	—	140

## 5. Comparison between thermochemical processes

All the previously discussed thermochemical conversion technologies have inherent limitations. They often produce lower-quality biofuels, involve high production and energy costs, and require extensive gas purification.^147^ Accordingly, understanding the advantages and limitations of each technology is essential for evaluating their performance, commercialization potential, and techno-economic feasibility.^5^ Unlike combustion, which mainly produces heat and electricity, pyrolysis offers a flexible route for liquid bio-oils and chemicals, where handling diverse feedstocks and moderate scales makes pyrolysis especially suitable for rural applications, providing opportunities for local biofuel generation and flexible energy solutions. However, it faces challenges such as poor bio-oil quality, limited catalyst life, scale-up issues, and the need for extensive upgrading.^148^ Conversely, gasification produces syngas that can be directly used as fuel or converted into valuable products, making it a versatile thermochemical conversion method. It also offers opportunities for large-scale operations and significant growth potential. However, gasification faces challenges, including high operating and maintenance costs, difficulties in recycling homogeneous and carbon-based catalysts, and high overall costs. HTL is a high energy efficiency method for converting wet biomass into bio-oil, making it particularly well suited for biofuel production. It eliminates the need for drying, and operates under relatively mild conditions, producing bio-oil with lower oxygen and moisture contents than pyrolysis oil. However, HTL faces challenges such as potential reactor corrosion, salt deposition, difficulties in recycling homogeneous or carbon-based catalysts, and variability in bio-oil properties depending on the feedstock and production conditions.^5^Table 10 summarizes the major advantages and disadvantages of thermochemical biomass conversion methods.^5,149^

**Table 10:** Advantages and disadvantages of thermochemical processes

Thermochemical method	Advantages	Disadvantages
Pyrolysis	-Flexible and scalable process suitable for diverse biomass feedstocks	-High costs associated with pre-drying biomass with high moisture content
	-Operates at low pressure with minimal feedstock pre-treatment	-Char formation can cause abrasion and erosion of equipment
	-Produces multiple valuable products: biochar, bio-oil, syngas, and hydrogen	-Syngas often requires extensive cleaning before use
		-Significant capital investment is needed
		-Catalyst deactivation due to char deposition
		-Feasibility is established only in large-scale plants
Gasification	-Achieves maximum biomass conversion	-Suitable for biomass with low moisture content; high-moisture feedstocks require pre-drying
	-Produces syngas and biochar	-Technology is complex with high capital and operating costs
	-Capable of high hydrogen yields	-Syngas requires extensive cleaning to remove tar, char, and ash
	-High efficiency even at small scales	-Feedstocks like manure can release H_2_S, necessitating additional gas cleaning
	-Flexible and scalable in capacity	
	-Can handle diverse biomass feedstocks efficiently to produce fuel gas	
Hydrothermal liquefaction	-Suitable for high moisture content biomass, thus drying is not required	-A high-pressure pump is needed to handle highly concentrated biomass feedstock and prevent reactor clogging
	-The product contains less oxygen and moisture than pyrolysis bio-oil	-Poor design may cause pipeline or reactor failure under high temperature and pressure
		-Corrosion and salt deposition possibility
Combustion	-Technology is simple and widely available	-Suitable for low-moisture biomass; high-moisture feedstocks require costly pre-drying, reducing efficiency
	-Low operating costs	-Generates by-products (soot, ash, NO_x_, CO, CO_2_) with potential environmental impacts
	-Minimal biomass pre-treatment required	-Flue gas cleaning is necessary

## 6. Artificial intelligence (AI) in biomass conversion

Artificial intelligence (AI) is a sophisticated approach applied widely in various disciplines. It has the advantages of avoiding the lengthy experimentation, rigorous calculations, and high cost. The basic concept of AI is to analyze data, pattern derivation, and accordingly generate predictions.^150^ AI models for biomass thermochemical processes are important tools for research and industry, as they can establish a connection among biomass composition, operating conditions, and biomass utilization.^151^ AI algorithms depend on large datasets for model development, which achieves accurate results and ensures the formation of products with the desired properties by optimizing the operating conditions.^152^ AI models have been effectively utilized to predict the outputs of biomass thermochemical conversion, as listed in Table 12.

The application of AI in the biomass feedstock stage is valuable for predicting the yields from climate and cultivation variables, detecting anomalies, and integrating with control systems to improve the quality and efficiency. In the bioenergy production stage, processes such as pyrolysis and hydrothermal liquefaction are energy intensive, but their dependence on controllable reaction parameters makes them suitable for AI-based optimization to enhance the bioenergy yield and overall process efficiency.^153^ Schmidt *et al.*^154^ applied Bayesian optimization to tune the hyperparameters of various AI models for predicting biomass gasification gases. The results showed that XGBoost delivered the highest accuracy and robustness. In the biofuel application stage, AI research has focused on linking biofuel properties such as cetane number, density, viscosity, and calorific value to engine performance, aiming to enhance the combustion efficiency and lower emissions.^153^ For instance, Yatim *et al.*^155^ proved that the ANN model is suitable for predicting the biodiesel density, offering guidance for engine developers on operating conditions and biodiesel selection. In addition to the contribution of AI to improving the biomass yields, it also reduces waste by predicting by-product volumes and compositions, enabling their reuse as feedstock in other processes. This supports circular economy practices by converting waste into valuable resources.^156^ Moreover, AI-driven demand forecasting is transforming green energy management in manufacturing by accurately predicting energy needs and aligning them with production schedules. This approach minimizes waste, reduces emissions, and strengthens the sustainability.^157^

The most frequently used machine learning (ML) models for thermochemical biomass conversion include artificial neural networks (ANNs), support vector machines (SVMs), decision trees (DTs), and random forests (RFs). ANNs have been the most prominent method for pyrolysis process modeling due to their ability to learn highly nonlinear relationships between inputs and outputs, making them well suited for capturing the complexity of the pyrolysis process. Likewise, in gasification, ANNs have also been the most widely used ML method.^158,159^ SVMs can handle classification and regression with low generalization error and minimal computing cost, using kernel functions for complex, nonlinear relationships. DTs recursively split data into a tree structure of root, decision, and leaf nodes, offering easy interpretation and handling of missing data but are prone to overfitting and poor generalization. RFs are ensembles of decision trees that improve accuracy and reduce overfitting by training multiple trees on bootstrapped datasets and random feature subsets, though they can be slow for real-time predictions with large numbers of trees. Other ML methods, such as extreme gradient boosting (XGBoost) regression and adaptive network-based fuzzy inference system (ANFIS), are also employed in biomass conversion.^159,160^ Ensemble methods such as gradient boosting regression (GBR) improve the prediction reliability by combining multiple models, each capturing different aspects of the data. Madadi *et al.*^161^ found that GBR was the most effective in optimizing the biphasic pretreatment conditions for lignocellulosic biomass fractionation. Another recent study by Qiao *et al.*^162^ showed that CatBoost Regressor outperformed the other seven evaluated models in optimizing the conversion of cellulose into 5-hydroxymethylfurfural. Table 11 lists the advantages and limitations of some ML models.

**Table 11:** Advantages and limitations of ML models used in thermochemical biomass conversion^158,159^

Model	Advantages	Limitations
ANN	Ability to learn highly nonlinear relationships between inputs and outputs	-Low precision, preprocessing needs, and overfitting risk
		-Incapable of explaining their reasoning or providing a basis for their decisions
SVM	Resist overfitting, have low generalization error, and perform efficiently on small datasets	Highly sensitive to kernel choice and parameter tuning
DT	Easy to interpret and handle missing or duplicate data	Prone to overfitting and poor generalization
RF	Handle large datasets and reduces overfitting	Too many trees slow real-time predictions despite fast training
XGBoost	-Precise loss calculation using first and second order derivatives	-Can overfit small datasets if not properly tuned
	-Reduce overfitting through regularization	-Computationally expensive, particularly on large datasets or with complex models

**Table 12:** Studies on AI models applied in biomass thermochemical conversion

Process	Biomass	Input	Output	Algorithm	References
Co-pyrolysis	52 distinct lignocellulosic biomasses and 55 plastic types	Process and feedstock characteristics	Biochar and liquid yields	CatB and XGB	163
Microwave-assisted co-pyrolysis	A variety of biomass feedstocks such as lignin, rice straw, *Chlorella vulgaris*, and plastic feedstock (LDPE, PS, and PVC)	Proximate and ultimate analyses of biomass and plastic, heating rate, pyrolysis temperature, proportion of biomass and plastic in the mixture	Biochar, bio-oil, and biogas yields	XGBoost	164
Pyrolysis	15 types of lignocellulosic biomass, 12 types of plants, and 6 types of algae	-Ultimate and proximate analyses	Bio-oil yield	-Random Forest	165
		-Heating rate, particle size, highest treatment temperature, and N_2_ flow rate		-Decision tree	
				-Support vector machine	
				-Multi linear regression	
Pyrolysis	Herbaceous plants, lignocellulosic biomass, sewage sludge, and animal waste	-Ultimate, proximate, and structural analyses of biomass	Biochar yield	ANN and metaheuristic algorithms	166
		-Pyrolysis conditions			
Torrefaction	Agricultural residues, forestry residences, and energy crops	-Ultimate and proximate analyses	The yield of solid products	Gradient tree boosting was the most accurate	167
		-Torrefaction conditions (temperature, residence time, (CO_2_, CO, N_2_ fractions in the reacting gas)			
Hydrothermal liquefaction	Pine	The carbon contents, reaction temperature, and biomass concentration	Biocrudes heating values	Support vector machine (SVM)	168
Acid-catalyzed liquefaction	Lignocellulosic biomass	Biomass composition, particle size, solvents, catalyst, and liquefaction conditions	Bio-polyols yield and heating value	GBR	169
Gasification	20 different biomass samples (agricultural and herbaceous waste)	Temperature, steam and fuel flow rates, and the content of H, O and C	Syngas exergy value	ANN and Aspen Plus® model	170
Gasification	Sugarcane bagasse	Biomass characteristics and gasification conditions	Syngas yield	XGBoost	171
Gasification	Lignocellulosic biomass	Laboratory and pilot plant experimental data	Gasification products	XGBoost	154

## 7. Bio-oil, biochar and pyrolysis gas characteristics

Bio-oil is a mixture of various molecular weight components produced from the thermal degradation of lignocellulosic materials. It features a dark-brown colored liquid and viscous texture consisting mainly of carbon, oxygen, and hydrogen. The major categories of bio-oil compounds encompass phenols, aromatics, aldehydes, ketones, esters, carboxylic acids, alkanes, and nitrogen-bearing compounds.^172^ Typically, bio-oil consists of water (20–30%), organics (20–30%), water-soluble pyrolytic lignin (28–36%), and water-insoluble pyrolytic lignin (15–23%).^173–175^ In addition to its potential as a biofuel source, bio-oil can be utilized as a feedstock for manufacturing plastics, fertilizers, pesticides, pharmaceuticals, carbonaceous materials, *etc.*.^12–14^ A recent study by Ucella-Filho *et al.*^176^ investigated the antiviral potential of *Citrus sinensis* bio-oil produced at different heating rates. One sample exhibited anti-SARS-CoV-2 activity, and the findings offer valuable guidance for future research on developing bioactive products from bio-oil. Another study by Abd-Elnabi *et al.*^177^ reported that olive cake bio-oil contains bioactive compounds with potential as biopesticides. An insect bioassay against aphids, conducted using the slide dip method, demonstrated significant mortality in these sucking pests. Bio-oil has the advantages of carbon neutrality and lower SO_*x*_ emissions. However, the direct application of bio-oil as a fuel substitute is hampered due to its high viscosity, high moisture content, low calorific value, elevated oxygen content (10–40%), and acidity (corrosiveness). Biochar is the solid material that remains after the thermochemical degradation of biomass in the absence or lack of oxygen (pyrolysis), which basically consists of carbon, in addition to hydrogen, oxygen, and ash. The composition of biochar varies depending on the biomass feedstock, pyrolysis temperature, and heating rate. Lignin-rich biomass yields a high fraction of biochar.^178,179^ As the pyrolysis temperature exceeds 400 °C, the surface area of the biochar begins to increase up about 900 °C, which is attributed to the release of volatiles inducing pores formation.^180,181^ Biochar has several applications, for instance, heat generation, soil remediation, adsorbent for air and water pollutants owing to its porous structure, flue gas cleaning, and biodiesel production catalyst.^178,179,182–184^ Packialakshmi *et al.*^185^ showed that coconut shell biochar can be used as an adsorbent for electroplating wastewater, effectively reducing the zinc and potassium concentrations. Moreover, biochar can function as an electrode material for energy production and energy storage devices due to its large surface area and conductivity.^186^ For instance, Zhang *et al.*^187^ used three biomass feedstocks (dairy manure, sewage sludge, and wood chips) to fabricate carbon electrodes. Their study showed that biochar can be converted into carbon electrodes, with their oxygen reduction reaction activity influenced by the feedstock type and pyrolysis temperature. Another recent study by Nambyaruveettil *et al.*^188^ developed and optimized a novel Ni-zeolite-biochar hybrid catalyst for green hydrogenation using mordenite zeolite and date pit biochar, presenting an innovative and eco-friendly flexible catalytic support. To utilize biochar in a broad range of applications, its surface area and functional groups need to be upgraded by applying several techniques such as physical and chemical activation, gas activation, and steam activation.^180,181,189,190^

The gas mixture produced during pyrolysis primarily consists of CO_2_, CO, H_2_, CH_4_, C_2_H_4_, and C_2_H_6_, along with smaller amounts of NH_3_, C_3_H_8_, sulfur oxides, nitrogen oxides, and low-carbon alcohols. Its typical energy content ranges from 10 to 20 MJ m^−3^. However, for practical applications, impurities such as aerosols, water vapor, NH_3_, HCN, and H_2_S must first be removed.^191^ The pyrolytic gas composition is influenced by factors such as temperature, residence time, and properties of the biomass feedstock used in the pyrolysis process.^192^ Light hydrocarbons, particularly CH_4_, originate from the decomposition of weak methoxyl (–O–CH_3_) and methylene (–CH_2_–) groups and the secondary breakdown of oxygenated compounds, while H_2_ is formed *via* secondary decomposition and reforming of aromatic C=C and C–H bonds at elevated temperature.^193^ However, the thermal degradation of the carbonyl and carboxyl groups in the three lignocellulosic components is thought to produce CO_2_ and CO.^194^ Pyrolytic gas can be utilized for heat and power generation in engines, as a self-feeding source in continuous pyrolysis reactors, or as a carrier gas in fluidized bed systems. However, its short combustion time, difficult storage, and thermal instability limit its efficient and long-term use.^191,194^ Yu *et al.*^195^ highlighted that syngas purification and CO_2_ capture can significantly enhance the efficiency and value of pyrolysis gas utilization, particularly if the captured CO_2_ is further synthesized. Another study by Straka *et al.*^196^ found that pyrolysis gas from apricot stones and walnut shells can be converted into H_2_-rich gas using a Pd/C catalyst, whereas CH_4_-rich gas can be produced using an Ru/Al_2_O_3_ catalyst in a sealed pressurized reactor, emphasizing that pressure-catalyzed biomass pyrolysis can efficiently produce energy-rich gas. A recent study by Brands *et al.*^197^ explored an innovative approach to convert pyrolysis gas into biomethane through a water-gas shift reaction and microbial methanogenesis.

### 7.1. Bio-oil upgrading

**Fig. 16:**
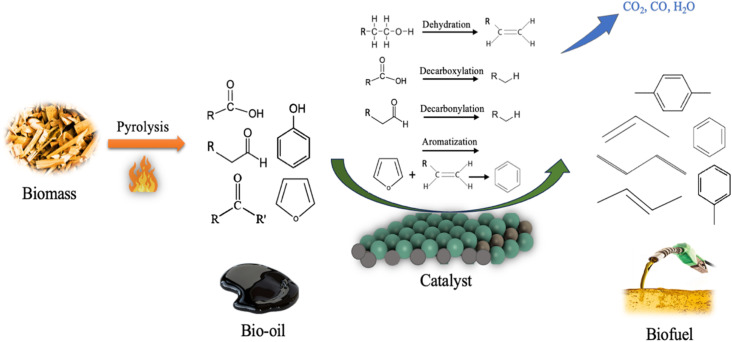
Major reaction pathways for bio-oil catalytic upgrading.

**Fig. 17:**
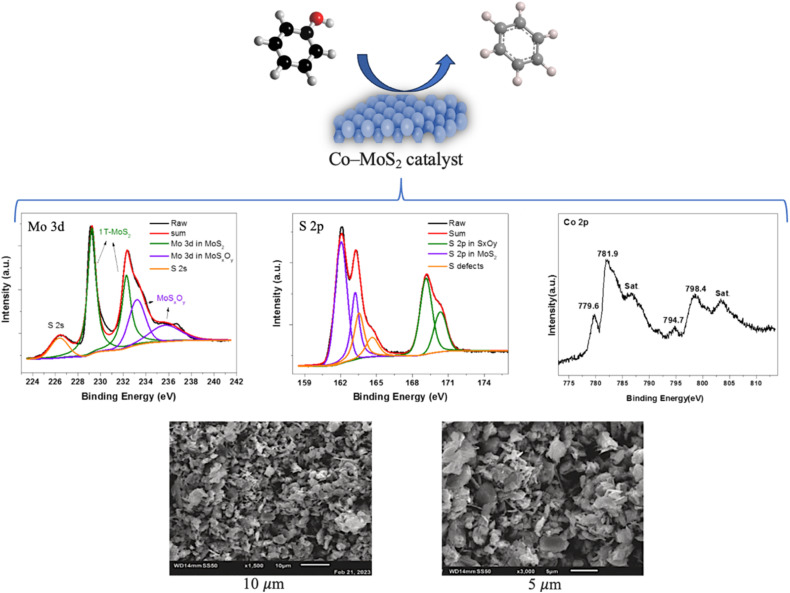
XPS spectra and SEM images of the Co-MoS_2_ catalyst, adapted from ref. 206 with permission from Elsevier, *Case Studies in Chemical and Environmental Engineering*, vol. 9, p. 100734, Copyright 2024.

Bio-oil upgrading is essential for its utilization as a fossil fuel alternative.^13,198,199^ The major bio-oil catalytic upgrading reaction pathways include dehydration, decarboxylation, and decarbonylation, as depicted in Fig. 16. Additionally, other reactions contribute to bio-oil upgrading such as aromatization, ketonization, cracking, and aldol condensation. Dehydration reaction takes place on the catalyst active sites, producing dehydrated products and water. Decarboxylation reaction expels oxygen from fatty acids in the form of CO_2_, and decarbonylation removes the carbonyl group from ketones and aldehydes, producing CO. Ketonization involves the reaction of two carboxylic acids to produce ketone, CO_2_, and water by coupling a C–C bond and removing one carboxyl group.^200,201^ The aromatization reaction for alkenes and low molecular weight oxygenated compounds such as furans produces aromatic hydrocarbon, CO_2_, CO, and water. This process can be interpreted through the hydrocarbon pool mechanism.^3,201^ Soldatos *et al.*^202^ investigated the *in situ* catalytic upgrading of beechwood lignin pyrolysates. Various aluminosilicate catalysts with different Si/Al ratios were employed, among which ZSM-5 (40) demonstrated a superior performance in producing BTX aromatics, while suppressing further condensation into PAHs. Chaerusan *et al.*^203^ investigated the catalytic upgrading of bio-oil from torrefied giant *Miscanthus* over copper-magnesium (Cu-Mg) bimetal-doped zeolites. The results showed a high yield of aromatic hydrocarbons (63%), predominantly favoring BTX compounds. Investigating the variation in surface electronic and structural properties during a specific process is crucial for developing new catalytic systems. X-ray photoelectron spectroscopy (XPS) is an analytical technique used for the surface species and valence analysis of catalysts; it is based on the photoelectric effect, which occurs when a material is exposed to high-energy photons, causing electrons emission.^204^ Scanning electron microscopy (SEM) is another analysis employed to explore the morphological characteristics of catalysts. XPS and SEM analyses have been employed in several studies,^205^ for instance Ismail *et al.*^206^ utilized the XPS and SEM techniques to explore the chemical state and morphology of a Co-MoS_2_ catalyst, as shown in Fig. 17. Similarly, Ali *et al.*^207^ investigated the characteristics of a 5%Ni-CeO_2_ catalyst *via* the aforementioned analyses.

Hydrodeoxygenation (HDO) is a catalytic upgrading process conducted under high hydrogen pressure (70–90 bar) and temperature (<400 °C), which aims to lower the oxygen level in bio-oil by reacting its oxygenated functional groups with hydrogen, acting as a reducing agent. HDO is considered the most efficient bio-oil upgrading treatment.^208,209^ ZSM-5 zeolite and sulfided CoMo and NiMo catalysts are commonly employed in hydrotreating bio-oil, where CoMo and NiMo are resistant to the sulfur content in the biomass feedstock.^210^ ZSM-5 and HZSM-5 zeolite catalysts show superior deoxygenation ability owing to their high acidity and accessible acid sites.^211^ Catalyst deactivation due to coke formation is the primary challenge in HDO treatment.^12^ Du *et al.*^212^ proposed a novel interfacial-enhanced HDO system for upgrading vanillin bio-oil. Hydrophobic silica was used as a solid emulsifier to construct a stable oil/water emulsion, enabling the interfacial HDO of vanillin with an Ni-based catalyst. This approach improved the conversion and increased the deoxygenated selectivity from 43% to 99% at 150 °C, demonstrating a superior performance compared to direct HDO. Xie *et al.*^213^ synthesized a series of low-loading Ni/C catalysts (1–7%) for the HDO of lignin bio-oil and phenolic compounds. Using 5% Ni/C at 200 °C for 2 h, guaiacol was completely converted to cyclohexanol, which can serve both as a source for chemical products and as a fossil fuel additive. Several studies related to bio-oil upgrading by HDO are summarized in Table 13. Besides HDO, there are several advanced bio-oil upgrading processes, as follows:

**Table 13:** Studies on HDO treatment

Biomass	Reactor	Catalyst	Main findings	References
Guaiacol as a model compound	Autoclave reactor	Ni-CoO_*x*_/Hβ-u catalysts	Under a hydrogen pressure of 1.0 MPa, guaiacol was completely converted to cyclohexane within 40 min at 180 °C	224
*Eucalyptus*-derived lignin oils	Autoclave	Mo/NiFe71-V	-The catalytic performance was influenced by Mo-Ni intermetallic interactions *via* charge transfer, with Mo/NiFe71-V showing excellent activity across a wide range of substrates	225
			-HDO of guaiacol at 240 °C achieved almost complete conversion, 99.5% cyclohexane yield, and 92.1% yield after six cycles	
Pine sawdust	Fluidized-bed reactor	Fe-Co/SiO_2_	22.44% was the highest HC content in the upgraded bio-oil	226
Wood	Autoclave	Rh, Pt, Pd on a zirconia support	37–47 wt% yield of biofuel	227
Loblolly pine	Auger pyrolysis reactor	Nickel/silica–alumina catalyst	HDO for the obtained bio-oil produced HC yield of 20.4 wt%	228
1,6-Anhydro-beta-d glucose	Tubular reactor	Ni-CeO_2_	Jet fuel fractions account for the highest share (47.0–68.0%), while gasoline components range from ∼9.0–22.9%	229
Acetone and phenol as model compounds	Autoclave	Raney Ni catalyst	The content of ketones and aldehydes reduced from 30.36% to 9.32%	230
Pine wood	Packed bed reactor	Ru/C and Pt/C catalysts	45% of the water-soluble carbon in the bio-oil was converted to gasoline blend stocks and C2 to C6 diols	231
Salicornia seeds	TGA	5 %Ni-CeO_2_	Jet fuel compounds yield ranged from 42–45% within temperature range 400–500 °C	207
Date palm pits	2-Stage pyrolysis reactor	Co-MoS_2_	Biomass was converted into non-oxygenated aromatics at temperature 500 °C	206
Date palm pits	Multi-stage catalytic pyrolysis reactor	Ni-doped-H-beta catalyst	BXT compounds (mainly toluene) peaked at 70% between 300–400 °C	232

• Solvent extraction by adding solvents such as methanol, ethanol, and acetone to reduce viscosity of bio-oil and enhance the heating values.^214^ Machado *et al.*^215^ assessed bio-oil deacidification *via* liquid–liquid extraction with aqueous methanol, showing that higher water and acid contents reduce the efficiency, while higher temperature enhances it. Another study by Zhao *et al.*^216^ evaluated the impact of different extraction solvents (acetone, ethanol, dichloromethane and ethyl acetate) on the recovery of bio-oil from rubberwood sawdust liquefaction in water, ethanol or water–ethanol mixed solvent. Among them, acetone achieved the highest oil yield with the water–ethanol solvent, while ethanol was the least effective.

• Emulsification by blending bio-oil with refinery distillates, with the aid of surfactants, to enhance the ignition characteristics.^217,218^ Bhat *et al.*^219^ emulsified bio-crude from the hydrothermal liquefaction of waste wheat flour and canola meal with light cycle oil (LCO) using octanol as an emulsifier. The resulting emulsion showed improved physicochemical properties, including acid number, density, viscosity, heating value, pour point, and elemental composition, compared to the raw bio-crude. Chong *et al.*^220^ produced bio-oil from palm kernel shells and developed a stable bio-oil/diesel emulsion *via* ultrasonic emulsification. Among the tested solvents (2-octanol, 2-heptanol, and 2-octanone), which were selected based on the computer-aided molecular design (CAMD) method, 2-octanol was identified as the most effective for stable emulsions. The optimal diesel:surfactant:bio-oil ratio was 80 : 15 : 5, preventing phase separation even at high temperatures. However, although promising, further evaluation of the fuel properties is needed for diesel engine applications.

• Supercritical fluids (SCFs) exhibit solvent properties that allow them to dissolve compounds that are not soluble in either the liquid or gas phase of the solvent. SCFs improve the calorific value and lower the bio-oil viscosity.^127,214^ Li *et al.*^221^ developed an eco-friendly supercritical CO_2_-ethanolysis system to convert *Caragana korshinskii* into valuable bio-oil. The process showed strong synergistic degradation, yielding 38.2 wt% bio-oil with a high heating value.

• Plasma processing generates reactive species, such as electrons, using high voltages (10–30 kV) and pulse frequencies (1–10 kHz), promoting the hydrodeoxygenation of bio-oil and improving the fuel quality. When combined with bifunctional catalysts or processes such as biomass-plastic co-pyrolysis, it enhances the H/C ratio. However, challenges including high costs, technical complexity, and catalyst deactivation from coke require further research.^222^ Fan *et al.*^223^ studied the catalytic pyrolysis of rapeseed shells using HZSM-5 coupled with plasma discharge, producing bio-oil with a high purity of monocyclic aromatic hydrocarbons and no detectable polycyclic aromatic hydrocarbons, and with its C_8_–C_12_ carbon range, the refined bio-oil is suitable as a gasoline or diesel additive.

## 8. Challenges and future prospects

Biomass valorization into fossil fuel substitutes through thermochemical conversion faces several challenges and presents prospects for further study. The high oxygen content in the produced bio-oil is the main challenge in biomass conversion, as it restricts its direct application as a fossil fuel substitute and leads to inferior quality compared to petroleum-based fuels.^1,233^ As discussed earlier, HDO is the most effective upgrading method as it increases the calorific value and enhances the fuel quality by reducing the oxygen content through reactions including cracking, decarbonylation, decarboxylation, and hydrogenation, with catalysts playing a key role.^234^ However, although noble metals such as Pt, Pd, Ru, and Rh show high activity in HDO, their high cost and scarcity limit their industrial application. Transition metals such as Ni, Fe, and Co, either alone or in combination and supported on suitable materials, can achieve comparable HDO performances. Among them, Ni is the most widely used due to its low cost, strong hydrogenation ability, and efficient C–O bond cleavage.^235^ A recent study by Wu *et al.*^236^ reported that Ni-V bimetallic zeolite catalysts effectively upgraded pine nut shell pyrolysis vapors *via in situ* HDO. The treatment improved the pH and lowered the heating value of the bio-oil, reduced its moisture content and density, lowered its O/C ratio, and efficiently reduced its content of oxygenated compounds. Catalyst deactivation remains a major challenge, primarily driven by carbon deposition, sintering, and poisoning of the active sites. These processes diminish the efficiency of HDO catalysts by lowering their available surface area and modifying their chemical properties.^234^ Designing nanocatalysts with a core–shell structure is a promising approach to enhance the catalyst stability, as it helps prevent nanoparticle migration and sintering.^237^ Deng *et al.*^238^ showed that an Ru@SiO_2_-ZrO_2_ core–shell catalyst outperformed conventional Ru/SiO_2_-ZrO_2_, maintaining its activity over five cycles without notable deactivation. This catalyst displayed an excellent HDO performance for phenolic compounds and lignin pyrolysis oil, increasing its hydrocarbon content from 12.7% to 96.9%, and thus highlighting its strong potential for industrial application. Catalysts often suffer deactivation from carbon deposition. Thus, common approaches such as thermal oxidation, steam treatment, and gasification are primarily employed to remove coke, poisons, or surface deposits, which diminish the catalyst activity. However, although widely used, these methods typically require significant energy and can potentially damage the structural integrity of the catalyst. Also, the conventional regeneration methods often fail to fully recover the catalyst activity, prompting the development of emerging techniques, such as plasma treatment and microwave-assisted regeneration to overcome the severe deactivation, structural damage, and loss of selectivity in catalysts.^239,240^ Non-thermal plasma (NTP) has emerged as an efficient alternative for catalyst regeneration, enabling non-thermal molecular activation and the generation of highly reactive species under ambient pressure and near-room temperature conditions.^241^ During NTP, the excited radicals oxidize and gasify coke on the catalyst at low temperatures, forming CO and CO_2_ without overheating the catalyst.^240^ Li *et al.*^242^ showed that direct NTP regeneration effectively restored the activity of deactivated HZSM-5 for upgrading rape straw pyrolysis vapors by removing coke and generating active sites, while also increasing the surface area, pore volume, and acidity of the catalyst.

Mechanical pretreatment is essential for most biomass conversion processes because biomass is typically a heterogeneous solid with varying sizes, shapes, and compositions, which can reduce the conversion efficiency and complicate handling and transport. Processes such as crushing, grinding, drying, and densification help produce a more uniform feedstock, improving the material handling and overall conversion performance.^90,243^ The particle size of biomass feedstock significantly impacts bio-oil production, influencing both the yield and quality. Fast pyrolysis favors smaller particles, which enable efficient and uniform heat transfer, whereas larger particles hinder heat penetration, causing lower internal temperatures, and consequently reduced liquid yields, and thus require additional mechanical energy for feedstock physical pretreatment.^244–246^ Biomass feedstocks for pyrolysis often contain over 40% moisture, necessitating pre-treatment such as atmospheric drying, solar drying, or evaporation. Although solar drying is economical, it is time-consuming. Also, the high water content in biomass increases the energy consumption for evaporation, thereby limiting the process efficiency and economic viability.^247^

Techno-economic analysis (TEA) is vital for evaluating the feasibility and sustainability of bio-oil production. It quantifies costs, profitability, and long-term viability. Given that fossil fuels remain cheaper, bio-oil must demonstrate cost competitiveness and economic sustainability to scale successfully. Its economic feasibility can be enhanced through flexible feedstock use, co-product valorization, and efficient upgrading methods that lower costs and improve the fuel quality.^69,148^ Additionally, it is crucial to optimize a system that can handle the variation in biomass feedstock materials. The inconsistency in biomass can be mitigated by developing standards for the feedstocks that can be defined based on parameters such as moisture content and LHV.^20,39^

The use of AI in biorefineries is limited by poor data quality and insufficient data quantity. Hybrid models, which combine data-driven and mechanistic approaches, reduce data needs, while advanced sensors improve data quality and availability. Soft sensors further address these gaps by estimating unmeasured variables in real time, enabling timely interventions and ensuring accurate outcomes.^156^ Current ML studies in thermochemical conversion mainly predict single bioenergy products, overlooking competitive reactions among biochar, bio-oil, and biogas. Thus, future research should address these interactions to optimize the yields and clarify the mechanisms.^153^

## 9. Conclusions

This study reviews the valorization of biomass materials into bioenergy through thermochemical processes (pyrolysis, gasification, liquefaction, and combustion). It can be inferred that biomass is a promising source for bio-oil, syngas, biochar, and heat, which can contribute into minimizing the reliance on fossil fuel sources and satisfy the global demand for renewable energy. Pyrolysis and hydrothermal liquefaction have demonstrated strong potential for converting lignocellulosic biomass into liquid bio-oils, which can be further upgraded into biofuels. The gasification process can be used to produce hydrogen, an emerging renewable energy source with applications such as fuel cell electric vehicles. Thermochemical techniques encounter several challenges that restrict their direct application as a sustainable and commercial energy source. The complexity of biomass feedstock complicates the optimization of systems able to treat the inconsistent feed. Moreover, the formation of oxygenated compounds, which reduce the fuel calorific value, calls the upgradation of bio-oil. HDO is the most effective upgrading technique. Nevertheless, the short lifetime of the catalyst due to its deactivation is the main challenge faced. Future research should prioritize emerging techniques such as non-thermal plasma and microwave-assisted regeneration to mitigate severe catalyst deactivation and associated structural damage. Additionally, developing nanocatalysts with a core–shell structure offers a promising strategy to improve the catalyst stability. Blending bio-oil with petroleum-based fuels through emulsification supports a more economical path for biofuel production. Further research should emphasize the integration of solar energy and biomass conversion technologies to tackle climate change and promote a shift toward cleaner energy sources. Although ML has shown success in bioenergy production, its use is still emerging and largely limited to lab-scale systems. Further research is needed to apply AI in the thermochemical valorization of biomass for its commercial and sustainable conversion.

## Conflicts of interest

There are no conflicts to declare.

## Data Availability

No primary research results, software or code have been included, and no new data were generated or analysed as part of this review.
